# Promoting Effect of Pinostrobin on the Proliferation, Differentiation, and Mineralization of Murine Pre-osteoblastic MC3T3-E1 Cells

**DOI:** 10.3390/molecules22101735

**Published:** 2017-10-16

**Authors:** Chengbo Gu, Linan Fu, Xiaohan Yuan, Zhiguo Liu

**Affiliations:** 1 Key Laboratory of Forest Plant Ecology, Ministry of Education, Northeast Forestry University, Harbin 150040, China; linan_fu@163.com; 2 Life Science and Biotechnique Research Center, Northeast Agricultural University, Harbin 150030, China; yuanxiaohana@163.com

**Keywords:** pinostrobin, MC3T3-E1, proliferation, osteogenic differentiation, osteoporosis

## Abstract

Pinostrobin (PI), a natural flavonoid found in a variety of plants, is well known for its rich pharmacological activities. However, its osteogenic function remains unclear. The aim of this study is to evaluate the effect of PI on the proliferation, differentiation, and mineralization of murine pre-osteoblastic MC3T3-E1 cells in vitro using MTT, alkaline phosphatase (ALP) activity, the synthesis of collagen I (Col I) assay, and Von-Kossa staining, respectively. The expression of osteocalcin (OCN) mRNA in cells was detected by real-time PCR. The effect of PI on the differentiation of dexamethasone (DEX)-suppressed cells was also investigated. The results showed that PI greatly promoted the proliferation of MC3T3-E1 cells at 5–80 μg/mL (*p* < 0.05 or *p* < 0.01), and caused a significant elevation of ALP activity, Col I content, and mineralization of osteoblasts at 10–40 μg/mL (*p* < 0.05 or *p* < 0.01), and the expression levels of OCN gene were greatly upregulated after PI treatment (*p* < 0.01). Furthermore, PI could rescue the inhibition effect of cell differentiation induced by DEX. Taken together, these results indicated that PI could directly promote proliferation, differentiation, and mineralization of MC3T3-E1 cells and has potential for use as a natural treatment for osteoporosis.

## 1. Introduction

Osteoporosis (OP) is a bone disease that is common in the aging population and among post-menopausal women, characterized by low bone mass and micro-architectural deterioration of bone tissue, rendering the individual highly susceptible to fragility fractures [1,2]. Causes of osteoporosis include estrogen deficiency, genetic disorder, nutritional deficiencies, chronic diseases, chronic exposure to medications, and aging [3].

Nowadays, with the extensive use of glucocorticoids, glucocorticoid-induced osteoporosis has become one of the most distinct and serious adverse effects associated with glucocorticoid therapy. Available therapies for osteoporosis include estrogens, selective estrogen receptor modulators, bisphosphonates, and calcitonin [4]. Hormone (estrogen) replacement therapy (HRT) has been shown to be effective in both preventing postmenopausal osteoporosis and decreasing fracture incidence [5]. However, HRT also increases the risk of breast and endometrial cancer [6,7]. Alternative and natural therapeutic strategies with minimal side effects are desperately required. It has been recently reported that some naturally occurring compounds induce MC3T3-E1 cell differentiation and have potential activity against osteoporosis [8,9,10,11]. It has also been reported that naringin, a plant-derived compound with a flavonone skeleton [12,13], has bioactivity against osteoporosis [14,15,16].

**Figure 1:**
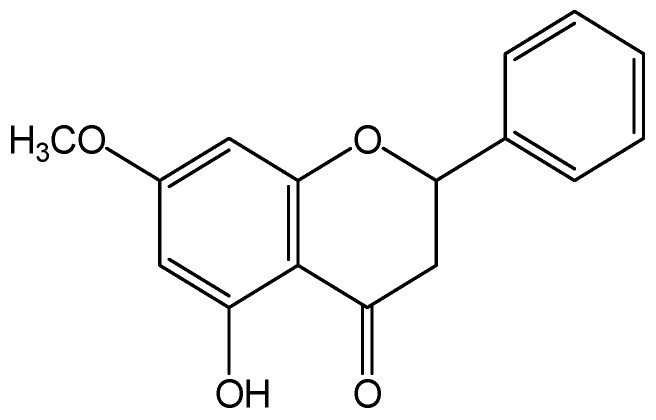
Chemical structural formula of pinostrobin (PI).

Pinostrobin (PI), 5-hydroxy-7-methoxy flavanone, is a naturally occurring flavonoid that exists in (*Cajanus Cajan* (L.) Millsp.), *Salvia texana* (Torr.), *Pinus strobus* (L.), *Boesenbergia pandurata* (Roxb.), and other plants. PI is known to exhibit diverse pharmacological activities including anti-oxidative [17], anti-inflammatory [18], anti-microbial [19], anti-virus [18,20], anti-Alzheimer [21], and anti-cancer [22,23] properties. In addition, PI has been found to have significant effects on inhibiting growth, arresting the cell cycle, and inducing apoptosis in many cancers and leukemia [22]. The structure of PI (shown in Figure 1) is similar with that of naringin. PI may carry out similar bioactivitiy with naringin. Recent research has found that water extract of *C. cajan* leaves is used to prevent and treat osteonecrosis of the femoral head in clinical practice in China [24]. PI is one of the main active constituents in *C. cajan* leaves [25]. Therefore, we hypothesized that PI may possess protective action against osteoporosis.

Osteoblasts are the bone-forming cells of the skeleton. They synthesize and regulate the deposition, maturation, and mineralization of the extra-cellular matrix of bone. The three principal periods of development are proliferation, matrix development and maturation, and mineralization. The pre-osteoblastic MC3T3-E1 cell is a well accepted model of osteogenesis for the study of osteogenic development in vitro [26].

The purpose of this work was to investigate the effects of PI on the proliferation, differentiation, and mineralization of osteoblastic cell lines in vitro. Furthermore, the effect of PI on the inhibition of osteogenic differentiation induced by DEX was also tested in this study.

## 2. Results

### 2.1. The Effect of PI on MC3T3-E1 Cell Proliferation

**Figure 2:**
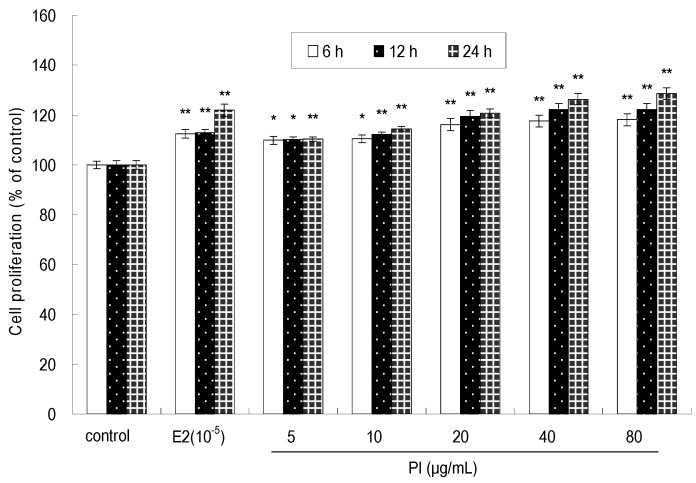
The effect of PI on the proliferation rate of MC3T3-E1 cells (* *p* < 0.05, ** *p* < 0.01 compared with the control group, *n* = 6).

As shown in Figure 2, the effect of PI at 5–80 μg/mL on cell proliferation was significant (*p* < 0.05 or *p* < 0.01) at 6 h, 12 h, and 24 h. PI promoted the proliferation of MC3T3-E1 cells in a time-dependent and dose-dependent manner. The maximum stimulatory effect on cell proliferation was achieved on 24 h when PI was at a concentration of 80 μg/mL. However, no significant stimulatory effect on the cell proliferation was observed after treatment with 40 or 80 μg/mL of PI at all assayed times points. As a positive control, 17-β-estradiol (E2) at 1 × 10^−5^ μg/mL stimulated MC3T3-E1 cell proliferation at 24 h, 48 h, and 72 h (*p* < 0.01). Moreover, the cell proliferation rate was higher in the groups treated with PI (20, 40 and 80 μg/mL) than that in E2-treated groups at 24 h, 48 h, and 72 h.

### 2.2. The Effect of PI on MC3T3-E1 Cell Differentiation

**Figure 3:**
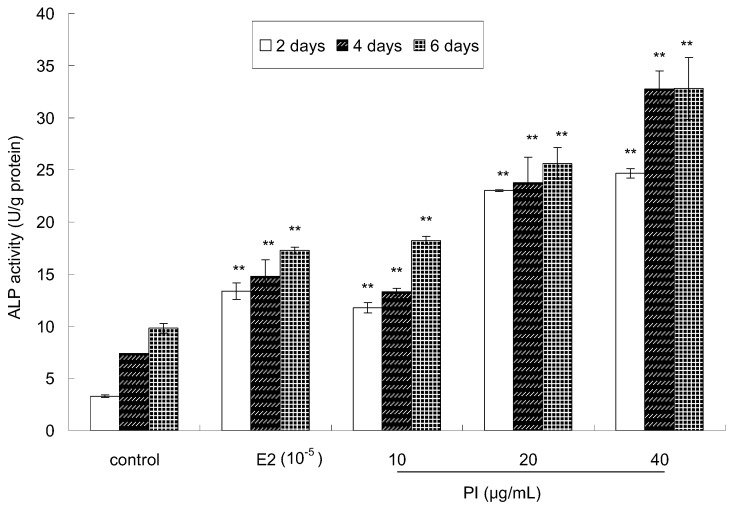
The effect of PI on the differentiation of MC3T3-E1 cells (** *p* < 0.01 compared with the control group, *n* = 6).

The activity of alkaline phosphatase (ALP), an early maker of osteoblast differentiation, was measured to investigate the effect of PI on the osteoblastic differentiation of MC3T3-E1 cells. The cells were treated with PI for 2 days, 4 days, and 6 days at various concentrations (10, 20 and 40 μg/mL). As shown in Figure 3, very significant differences (*p* < 0.01) were observed between the PI-treated group and the control group at all assayed time points. Moreover, ALP activity was higher in the PI-treated group than that in the control group, which showed a dose-dependent manner. ALP activity was also higher in the E2-treated group than that in the control group. Thus, these results indicated that PI could promote osteoblastic cell differentiation.

### 2.3. The Effect of PI on the Col I Content of MC3T3-E1 Cells

**Figure 4:**
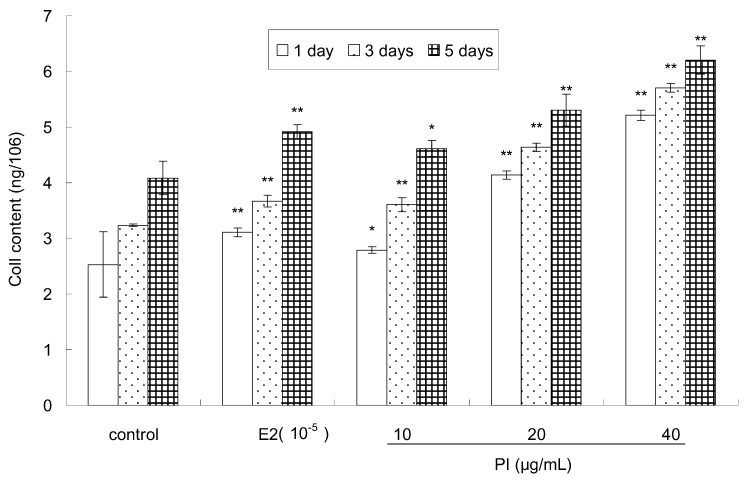
The effect of PI on the Col I content of MC3T3-E1 cells (* *p* < 0.05, ** *p* < 0.01 compared with the control group, *n* = 6).

Col I, a major protein in the bone matrix, is synthesized by osteoblasts and is involved in differentiation [27]. The effect of PI on the Col I content of MC3T3-E1 cells is shown in Figure 4. Compared to the control, the Col I content of MC 3T3-E1 cells was significantly elevated in a dose-dependent and time-dependent manner after treatment with various concentrations of PI (10, 20 and 40 μg/mL). E2 treatment also significantly increased the Col I content of MC 3T3-E1 cells. The maximal Col I content in MC3T3-E1 cells was observed at 5 days after treatment with 40 µg/mL of PI. Moreover, the Col I content was higher in groups treated with 20 and 40 μg/mL of PI than that treated with E2 at different assayed time points.

### 2.4. The Effect of PI on Osteoblast Mineralization of MC3T3-E1 Cells

**Figure 5:**
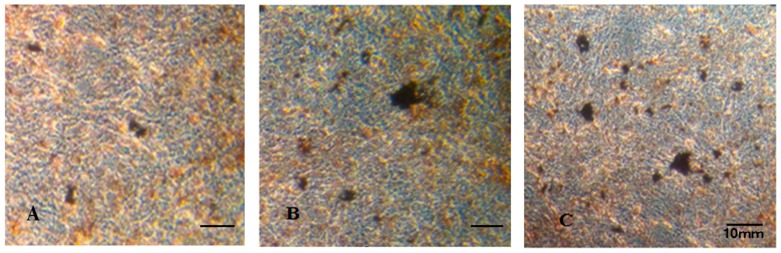
The effect of PI on the matrix mineralization of MC3T3-E1 cells after being cultured in osteogenic media for 28 days. The images showed Von Kossa staining in MC3T3-E1 cells and the scale bar represents 10 mm. ((**A**) control group; (**B**) E2 group; (**C**) PI (10 μg/mL) group).

The effect of PI on mineralization was determined via Von-Kossa staining. As shown in Figure 5, the calcified nodules appeared brownish-black in color by Von Kossa staining. Much more mineral deposition was found in the PI-treated (Figure 5C) and in the E2-treated group (Figure 5B) than that in the control group (Figure 5A).

**Figure 6:**
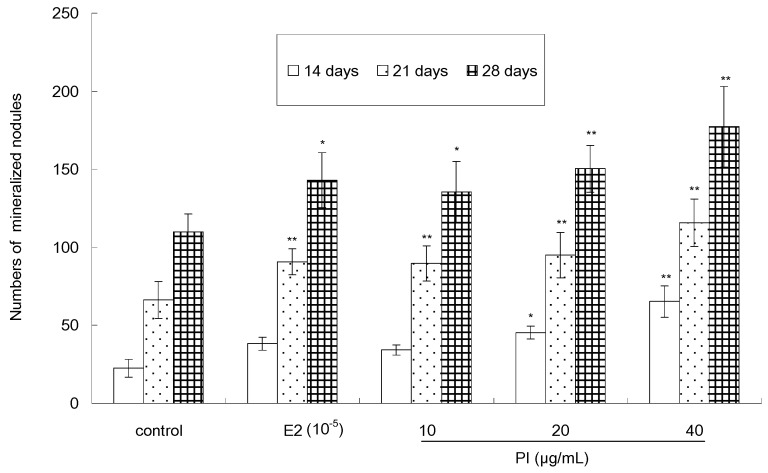
The effect of PI on numbers of mineralized nodules of MC3T3-E1 cells (* *p* < 0.05, ** *p* < 0.01 compared with the control group, *n* = 3).

The quantitative analysis result of Von-Kossa staining, as showed in Figure 6, indicated that PI (10, 20 and 40 µg/mL) could stimulate the formation of calcified nodules in a dose-dependent and time-dependent manner. The number of calcium nodules in the PI-treated group (except the cells treated with 10 μg/ mL of PI for 14 days) was significant (*p* < 0.05 or *p* < 0.01) than that in the control group. The number of calcified nodules reached its maximum when cells were treated with 40 μg/mL of PI for 28 days. As a positive control, E2 was able to increase the number of calcified nodules, as compared to the control. Furthermore, the stimulative activity of PI (20 or 40 µg/mL) was more potent than that of E2.

### 2.5. Expression Levels of OCN mRNA

**Figure 7:**
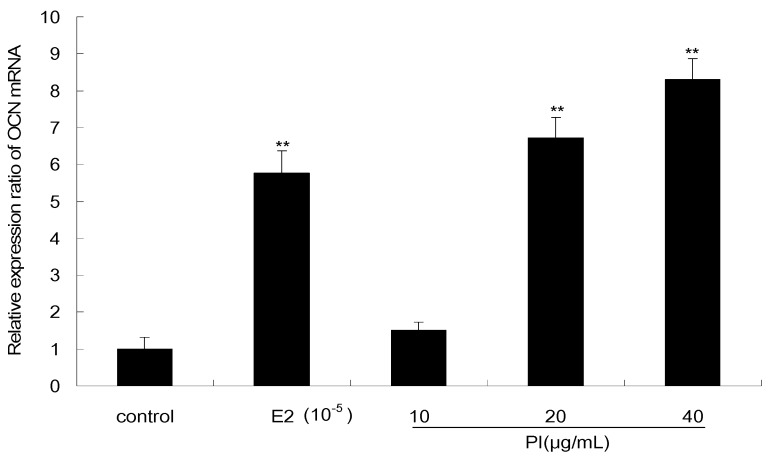
The effect of PI on the expression level of OCN mRNA in MC3T3-E1 cells (The results were normalized by the mRNA level of GAPDH. All experiments were performed in triplicate. ** *p* < 0.01 vs. the control group).

As shown in Figure 7, the effect of PI on the expression level of OCN mRNA in MC3T3-E1 cells was examined by quantitative real-time PCR. PI at concentrations of 20 and 40 μg/mL significantly increased the expression of OCN mRNA (*p* < 0.01), which were respectively upregulated by 6.7- and 8.3-fold as compared to the levels in the control. The OCN mRNA level was also significantly increased after E2 treatment (*p* < 0.01), which is upregulated by 5.7-fold as compared to the level in control.

### 2.6. The Effect of PI on DEX-Induced Inhibition of Osteogenetic Differentiation

**Figure 8:**
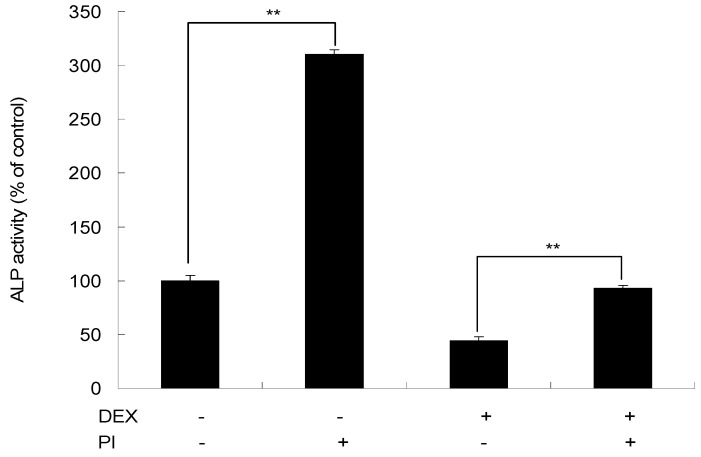
The rescue effect of PI on the inhibition of cell proliferation induced by DEX. MC3T3-E1 cells were treated with 10^−6^ M DEX, 40 μg/mL of PI or a combination of 10^−6^ M DEX and 40 μg/mL of PI for 4 days, and then ALP activity was determined. All experiments were performed in triplicate and ** *p* < 0.01. (The values in cells receiving neither PI nor DEX were set as 100%).

As shown in Figure 8, in the presence of DEX, there was a 55.74% decrease in ALP activity at day 4. After treatment with a combination of DEX and PI, the ALP activity was increased by 48.83% than DEX treatment alone (*p* < 0.01), which indicated the rescue effect of PI on the inhibition of cell proliferation induced by DEX.

## 3. Discussion

Natural products are a major source of drugs for the prevention or treatment of various disease. Isoflavonoids like genistein and daidzein extracted from plants have been confirmed to fight osteoporosis and promote bone health [28,29]. It has been demonstrated that isoflavones have a stimulatory effect on osteoblastic bone formation and an inhibitory effect on osteoclastic bone resorption, therefore increasing bone mass [30]. However, few studies that describe the anti-osteoporosis activity of botanical flavonone have been conducted. Naringin is a naturally occurring compound found in Fortune’s Drynaria Rhizome, grape fruits, and citrus fruits [12,13]. Studies have reported that naringin antagonizes osteoporosis [14,15,16], which may exert estrogen-like effects by promoting osteoblastic functions and suppressing osteoclastogenesis [31].

PI belongs to the flavanone subclass of flavonoids and has a structure similar to that of naringnin. These two structurally similar molecules may have similar biological activity. The current study we conducted further confirmed the osteogenic function of PI.

To clarify the role of PI, in bone formation and growth, the effects of PI on the proliferation, differentiation, and mineralization of osteoblastic cell lines were investigated using MC3T3-E1 in vitro. The MC3T3E1 cells have been reported to retain the capacity to differentiate into osteoblasts, which undergoes a temporal pattern of osteoblast development similar to in vivo bone formation [26,32].

The effect of PI on MC3T3-E1 cell proliferation was determined by an MTT method. The previous report showed naringin at 2–20 μg/mL exhibited slight but not significant proliferative activity in the MC3T3-E1 cells as compared to the control [12]. In our study, PI at 5–80 μg/mL significantly promoted the proliferation of the cells at 24 h, 48 h, and 72 h in a dose- and time-dependent manner (*p* < 0.05 or *p* < 0.01). Furthermore, the cell proliferation effect of PI at 40 or 80 μg/mL is stronger than that of E2 of 10^−5^ μg/mL at all assayed time points.

ALP and Col I are markers of the early period of osteoblastic differentiation [33,34]. It was reported that osteoblasts produce Col I and ALP, which are associated with matrix maturation and mineralization [35]. Results from the effect of naringin (2, 10, 20 μg/mL) on the ALP activity of MC3T3-E1 cells showed that only naringin at 2 μg/mL significantly improved the ALP activity of osteoblastic cells after 5 days of treatment [12]. In our study, the ALP activity was significantly enhanced after cells were exposed to PI at 10–40 μg/mL for 2 days, 4 days and 6 days. E2, an estrogen compound, acting as an antiresorptive agent, has been reported to enhance proliferation and differentiation of osteoblasts [36]. In this study, the ALP activity of cells exposed to PI at 20–40 μg/mL was higher than that of the cells exposed to E2 of 10^−5^ μg/mL at different tested time points.

The Col I content was also significantly increased by the treatment of 10–40 μg/mL of PI. Moreover, the Col I content of cells treated with PI at 20–40 μg/mL is higher than that treated with E2 of 10^−5^ μg/mL at different assayed time points. The formation of calcified nodule is a late marker of differentiation of osteoblastic maturation, signifying complete osteogenic differentiation [37,38]. In our study, more mineralized depositions were found in the PI-treated group than the control group, and the formation of calcified nodules was significantly stimulated in cells exposure to PI at 20 or 40 µg/mL for 14 days, 21 days, and 28 days as well as PI at 10 µg/mL for 21 days and 28 days, in a dose- and time-dependent manner.

OCN is another marker that expresses later during differentiation. We also examined the expression of the OCN gene in response of PI treatment. Previous evidence showed that the expression of the gene OCN increased by around 1.3-fold, as compared to that in control, after MC3T3-E1 cells were treated with naringin at 2 μg/mL for 4 days [12]. In our study, we found that the expression of this marker gene was upregulated by 6.7- and 8.3-fold, respectively, as compared to the control after cells were treated with PI at 20 and 40 μg/mL for 4 days. In addition, the OCN mRNA expression level of cells treated with PI at 20 or 40 μg/mL was 1.2- to 1.4-fold higher than that of cells treated with E2 of 10^−5^ μg/mL.

Glucocorticoids are widely applied as anti-inflammatory and immunosuppressive drugs for the treatment of many diseases and are one of the most frequent causes of secondary osteoporosis [39]. It has been reported that high concentrations of glucocorticoids inhibit bone growth, and that DEX (10^−6^ M), a synthetic glucocorticoid hormone, decreased ALP activity in MC3T3-E1 cells [40,41]. However, in our study, in the presence of PI, such inhibition was alleviated, which suggested that PI could ameliorate osteogenic differentiation impairment induced by DEX to some degree.

As a measure of the magnitude of a difference, effect size was also calculated, and we found that each eta square with *p* = 0.05 for each significant one-way analysis of variance in this study is greater than 0.95, which means that over 95% of the total variance is accounted for by the treatment effect. Almost all the variability came from the treatment effect. Therefore, there is a significant difference between the investigated groups.

A recent study showed that PI is non-toxic and non-genotoxic to male Wistar rats at higher doses of 500 mg/kg, and the LD50 of PI was found to be more than 500 mg/kg [42]. PI has many applications in dietary supplements marketed by Canadian Natural Herbal Products in the United States [43]. PI may be a promising agent for the prevention or treatment of osteoporosis.

## 4. Materials and Methods

### 4.1. Materials

PI, 17-β-estradiol (E2), dimethylsulfoxide (DMSO), methylt thiazolyl tetrazolium (MTT), dexamethasone (DEX), and trypsin were purchased from Sigma Chemical Co. (St. Louis, MO, USA). Fetal bovine serum (FBS), alpha-minimum essential medium (α-MEM), and penicillin-streptomycin antibiotic mixture were obtained from Hyclone (Hyclone, Logan, UT, USA). An alkaline phosphatase (ALP) activity kit was obtained from the Nanjing Jiancheng Biological Engineering Institute (Nanjing, China). A protein assay kit was purchased from Bio Rad. Trizol and SuperScript^®^ III Reverse Transcriptase were purchased from Invitrogen (Invitrogen, San Diego, CA, USA). A SYBR green kit for RT-PCRs was obtained from TaKaRa Biotech Co. (TaKaRa, Dalian, China). PI and E2 were dissolved in DMSO to make stock solutions, which were further diluted to different working concentrations with the medium before use (the final concentration of DMSO used in the culture was 0.01%).

### 4.2. Cell Culture

Mouse pre-osteoblastic MC3T3-E1 cells were obtained from China Center for Type Culture Collection (CCTCC) (Wuhan China). MC3T3-E1 cells were cultured in α-MEM containing 10% FBS and 1% penicillin-streptomycin (Hyclone, Logan, UT, USA) in a humidified, 95% air, 5% CO_2_ atmosphere at 37 °C.

### 4.3. Cell Proliferation Assay

MC3T3-E1 cells were suspended in medium and seeded at 2 × 10^5^ cells/well in 96-well plates. After 24 h of incubation, cells were treated with various concentrations of PI (5, 10, 20, 40 and 80 μg/mL) or with 10^−5^ μg/mL of E2 as a positive control for 6 h, 12 h and 24 h, respectively. Cells treated with the medium contained 0.01% DMSO were used as control, and wells without cells were set as blanks. On the indicated day of culture, 20 μL of MTT (5.0 mg/mL) was added and incubated for 4 h. Then, the supernatant was removed and 200 μL of dimethyl sulfoxide (DMSO) was added. After 15 min, the absorbance value (OD) was measured on a microplate spectrophotometer (Bio-Rad Model 680, Hercules, CA, USA) at 570 nm. The proliferation rate (%) was calculated using the formula: ODsample/ODcontrol × 100.

### 4.4. Cell Differentiation

MC3T3-E1 cells were plated in 12-well plates at a density of 5 × 10^5^ cells/well and were allowed to reach 80% confluence. The culture medium was then changed to α-MEM plus 5% FBS medium containing various concentrations of PI (10, 20 and 40 μg/mL) or with 10^−5^ μg/mL of E2 as a positive control. The cells were further cultured 2 days, 4 days, and 6 days. Cells treated with the medium contained 0.01% DMSO were used as control, and wells without cells were set as blanks. On the indicated day of culture, the medium was removed and the cell monolayer was gently washed thrice by ice-cold PBS and lysed with 100 μL of lysis buffer (0.2% Triton X-100). The lysate was centrifuged at 12,000 rpm for 5 min. The clear supernatant was harvested for the assay of ALP activity using the ALP Assay kit. Protein concentration of the cell lysate was determined by the Protein Assay kit.

### 4.5. Col I Content

MC3T3-E1 cells were plated in 12-well plates at a density of 5 × 10^5^ cells/well and were allowed to reach 80% confluence. The cells were then treated with various concentrations of PI (10, 20, and 40 μg/mL) or with 10^−5^ μg/mL of E2 as a positive control for 1 day, 3 days, and 5 days. After the exposure period, cells were harvested and suspended with 200 μL of saline. The suspensions were sonicated for 3 cycles of 5 s pulses with 10 s intervals at 75% amplitude. The disrupted cells were centrifuged at 3000 rpm for 5 min at 4 °C, and the resulting supernatants were analyzed for Col I content with an ELISA assay kit (Nanjing Jiancheng Biological Engineering Institute, Nanjing, China) as per the manufacturer’s instruction, and the absorbance was measured on a microplate reader at a wavelength of 450 nm.

### 4.6. Mineralized Assay

MC3T3-E1 cells were seeded in 6-well plates at a density of 1 × 10^5^ cells/well and cultured until 80% confluence. The cells were then treated with differentiation medium containing 10 mmol/L β-glycerophosphate and 50 μg/mL ascorbic acid (Sigma) with various concentrations of PI (10, 20 and 40 μg/mL) or with 10^−5^ μg/mL of E2 as a positive control for 14 days, 21 days, and 28 days, respectively. Every treatment was made in triplate. The formation of mineralized matrix nodules was assessed using Von-Kossa staining. In brief, the cells were rinsed twice with cold saline and were fixed with 4% formaldehyde at 4 ℃ for 30 min. The fixed cells were then rinsed twice with saline, followed by an addition of 5% (*w*/*v*) silver nitrate solution and the subsequent exposure to UV-light for 1 h. The cells were then stained with 5% (*w*/*v*) sodium thiosulfate solution for 5 min and counterstained with natural red. The presence of mineralized calcium deposits was qualitatively confirmed by brownish-black color staining, which was readily observable under bright-field light microscopy. Quantitative assessment of Von-Kossa staining was made by counting of stained nodules.

### 4.7. Quantitative Real-Time PCR

To analyze gene expression, the cells were plated in 6-well plates at a density of 2 × 10^5^ cells/mL and cultured in MEM containing 10% FBS until they reached 80% confluence. The cells were then treated with various concentrations PI (10, 20 and 40 μg/mL) and E2 of 10^−5^ μg/mL (as a positive control) for 4 days. At the end of the treatment time, cells were harvested and the total RNA was extracted using Trizol reagents. Reverse transcription of total RNA was conducted for 50 min at 42 °C and then 15 min at 70 °C, using SuperScript^®^ III Reverse Transcriptase. Following cDNA synthesis, quantitative RT-PCR was performed on an ABI 7500 Real-Time PCR System (Applied Biosystems Foster City, CA, USA) with SYBR Premix Ex Taq™ II according to the manufacturer’s instructions. The mRNA value for the OCN gene was normalized relative to the mouse GAPDH mRNA level in the RNA sample. The primers were designed as follows: OCN (forward primer, 5′-TGGCTTCTCTCCCTACTCCA-3′; reverse primer, 5′-GCAGCTGCAAAATCTCCTC-3′), GAPDH (forward primer, 5′-TCACCATCTTCCAGGAGCGA-3′; reverse primer, 5′-CACAATGCCGAAGTGGTGGT-3′). The PCR conditions were 95 °C for 3 min followed by 95 °C for 30 s and 58 °C for 40 s for a total of 35 cycles. All of the reactions were run in triplicate and the results were analyzed by the 2^−ΔΔ*C*T^ method.

### 4.8. Effect of PI on DEX-Induced Inhibition of Osteogenetic Differentiation

Cells cultured in 96-well plate were divided into four groups: the DEX group, where cells were treated with 10^−6^ M DEX; the DEX/PI group, where cells were treated with a combination of 10^−6^ M DEX and 40 μg/mL of PI; the PI group, where cells were treated with 40 μg/mL of PI; and the control, where cells treated with α-MEM supplemented with 5% FBS were used as a control. After 4 days of treatment, ALP activities of cells in different groups were measured by the method previously described.

### 4.9. Statistical Analysis

All data are expressed as mean ± standard deviation (SD). One-way ANOVA statistical analysis was conducted followed by a Tukey’s test for multiple comparisons if necessary. In all cases, *p* < 0.05 was considered significant, and *p* < 0.01 was considered as highly significant.

## 5. Conclusions

In summary, we demonstrated that PI can promote the proliferation, osteogenic differentiation, and mineralization of MC3T3-E1 cells. Therefore, PI may be a new promising candidate drug for the prevention or treatment of osteoporosis.

## Abbreviations

PI	pinostrobin
Col I	Collagen I
ALP	alkaline phosphatase
OCN	osteocalcin
DEX	Dexamethasone
